# The Levels of Zinc and Molybdenum in Hair and Food Grain in Areas of High and Low Incidence of Esophageal Cancer: A Comparative Study

**DOI:** 10.5539/gjhs.v4n4p168

**Published:** 2012-06-25

**Authors:** S. S. Ray, D. Das, T. Ghosh, A. K. Ghosh

**Affiliations:** 1 Department of Radiation Oncology, CCWH & RI, Calcutta, India; 2 Department of Radiation Oncology, University of Witwatersrand, South Africa; 3 Department of Instrumentation Sciences, Jadavpur University, Kolkata, India; 4 School of Oceanographic Studies, Jadavpur University, Kolkata, India; 5 Department of Instrumentation Sciences, Jadavpur University, Kolkata, India

**Keywords:** esophageal cancer, molybdenum & zinc, hair & food grain, South Africa, India

## Abstract

The outcome of different studies on the role of Zn & Mo in esophageal cancer (EC) is conflicting. Here, the levels of those elements in hair as well as food grain of two different ethnic populations across two continents have been studied to explore their role in EC. Two different ethnic populations are taken from (i) Eastern Cape, South Africa (RSA), an area of very high incidence of EC and (ii) West Bengal, India, an area of low incidence of that disease. Each ethnic population is divided into two groups: case and control (n=30 for all groups). Hair samples from all groups and food grains from RSA and India are analyzed for Zn & Mo content. This study shows a strong correlation between reduced levels of those elements in hair and the development of EC in RSA (both Zn & Mo: p<0.0001), though it is only suggestive in Indian context (both Zn & Mo: p≥0.05). Interestingly, control group of RSA shows significantly reduced level of those elements in hair even with respect to Indian case group (Zn: p<0.001 & Mo: p<0.00001). Food grain from RSA has significantly reduced level of those elements with respect to India (both Zn & Mo: p<0.0001). This deficiency of Zn & Mo in food grains can be correlated to the deficiency of those elements in hair of RSA population. The deficiency of Zn & Mo can be correlated to the development of EC.

## 1. Introduction

In South Africa, esophageal cancer (EC) is the third most common cancer in males (incidence is 5.4% of all cancers) but the incidence is moderately high among black males (17.14% of all cancers) (Sitas, Terblanche, & Madhoo, 1996). Worldwide, one in 32 men have a lifetime risk of developing EC while in Eastern Cape, South Africa it is twenty times more common that the second common cancer (Halperin, Perez, & Brady, 2008; Sitas et al., 1996). As per the National Cancer Registry, RSA, 1991, 3962 new cases of EC were reported in males and 1943 in females (Sitas et al., 1996). The incidence of EC in RSA is 12.24/100000 (crude rate) and 20.29/100000 (ASIR: age standardized incidence rate) for males and 5.4/100000 (crude rate) and 7.67/100000 (ASIR) for females. The incidence among the black males is 13.6/100000 and among black females is 5.9/100000 (Sitas et al., 1996). For black males the incidence rate is 13.07/100000 (crude rate) and 25.17/100000 (ASIR). As a whole in Eastern Cape, RSA 25% of all cancers is EC, whereas in Transkei, Eastern Cape, it is 50/100000 (ASIR) for both males and females (Sitas et al., 1996). The other global hot spots for EC are Linxian Province, China and Golestan Province in north eastern Iran (Halperin, Perez, & Brady, 2008). In eastern India in the state of West Bengal it has been found that the incidence of EC is 3.1/100000 and 2.9/100000 (crude rate) for males and females respectively (Indian Council for Medical Research [ICMR], 2000), which is much lower in comparison to that of Eastern Cape, South Africa or other global hotspots.

The role of Zn & Mo has been studied by many investigators. Some studies from endemic area of EC reported that plasma zinc was significantly reduced in EC compared to age matched healthy controls (Mellow et al., 1983; Barch & Iannaccone, 1986). In China, trace elements in hair and food grains were analyzed from high and low-incidence areas and an inverse correlation was found between EC mortality rate and the contents of molybdenum and zinc in hair and food grain samples (Yang, 1980). An inverse correlation between the development of EC and the contents of Zn & Mo in blood, hair and nail was reported from different studies (Nouri et al., 2008; Ahmad, Ghani, Azam, Bashir, & Begum, 2011). Some studies, using rat as experimental model have shown that zinc deficiency is one of the major factors associated with the increased risk of developing esophageal cancer (Barch, Fox, Rosche, Rundhaugen, & Wrighton, 1992; Fong & Magee, 1999) and zinc replenishment reduced esophageal tumor incidence in zinc-deficient rats (Fong, Farber, & Magee, 1998). Zinc also inhibits the formation of tumors and dietary zinc deficiency was also found to increase the incidence of tumours (Barch et al., 1986; Liang et al., 1999). The levels of zinc in tumors and normal tissues have been shown to be different (Margalioth, Schenker, & Chevion, 1983; Song, Heng, Rolandelli, Ament, & Heng, 1993; Ellwood, Roebuck, & Hathcock, 1994) and cell growth was shown to be affected by the applied zinc dosage (Ellwood et al., 1994; Prasad, 1979). Rensburg et al. (1980) found that minimal levels of zinc intake which ensure optimal body growth rate are inadequate to provide maximal resistance to esophageal carcinogenesis. Case control dietary study done in Washington, USA showed similar results that Zn deficiency can lead to EC (Rogers, Thomas, Davis, Vaughan, & Nevissi, 1993).

Several retrospective and prospective studies on humans and animals (Chan, Gerson, & Subramanium, 1998; Fong, Sivak, & Newberne, 1978; Waalkes, Kovatch, & Rehm, 1991; Birch & Fox, 1987) have shown that deficiency in trace elements such as Zn & Mo causes changes in enzymatic pathways producing carcinogenic end products like “nitrosamine and methylbenzylnitrosoamine” (MBN) resulting in EC (Barch et al., 1986; Chen, Cole, Mi, & Xing, 1992; Fong & Newberne, 1978). A deficiency of molybdenum has also been cited as a possible factor in the causation of EC (Yang, 1980; Luo et al., 1989).

Some studies have indicated that dietary deficiency of Zn is associated with the development of EC but the role of other trace elements like Mo are not conclusive (Chen et al., 1992; Barch, 1989). Conflicting results have also been reported from China. In one study (Dawsey et al., 1994), the Dysplasia Trial, it has been found that people with dysplasia of the esophagus do not do better even with vitamin and mineral supplements. In another study, the General Population Trial in the same area, it was found that patients with previously diagnosed precancerous lesions who were on Vitamins and mineral (containing Zn & Mo) did not have a reduced invasive lesions or mortality rates (Blot et al., 1993).

However, it is found that outcome of different studies are conflicting and the literature in this field are also scanty indeed. So, the role of trace elements (Zn & Mo) in the etiology of EC needs to be explored. Zn & Mo are deposited in hair and one of the sources of these elements is food grains consumed. In the present work the role of Zn & Mo in the etiology of EC has been studied by analyzing the amount of Zn & Mo both in food grain and hair.

## 2. Materials and Methods

Epidemiological studies indicate that Eastern Cape, South Africa is one of the hot spot and West Bengal, India is one of the cold spot for EC. That is why Indian population from West Bengal has been taken for this study.

The study included the following groups:

### 2.1 RSA (Eastern Cape) Population

#### 2.1.1 Case Group

Thirty EC patients are taken from the Surgical Department of Frere Hospital, Eastern Cape, South Africa. They are all above 18 years age, from black ethnical background and have squamous cell carcinoma of the esophagus. They have resided for more than 15 years in Eastern Cape, South Africa

#### 2.1.2 Control group

Thirty self volunteered people from the general population are taken. They are all above 18 years, from black ethnicity and do not have any history of EC. They have resided for more than 15 years in Eastern Cape, South Africa.

### 2.2 Indian (West Bengal) Population

Case group: Thirty EC patients are taken from the Surgical Department of CCWHRI Hosp, Kolkata, India. They are all above 18 years age, from Indian ethnical background and have squamous cell carcinoma of the esophagus. They have resided for more than 15 years in West Bengal, India.

Control group: Thirty self volunteered people from the general population are taken. They are all above 18 years, from Indian ethnicity and do not have any history of EC. They have resided for more than 15 years in West Bengal, India.

Inclusion criteria for all case groups:

a) Both males and females are above18 years.
b) Their staple dietary source is from local crops grown (at least a part of their diet was from the local crops grown).
c) The weight of all the subjects is above 36 kg.
d) The patients are able to swallow at least a liquid diet.

Control Group is taken from volunteered participation of the respective population of the respective study area.

Inclusion criteria for all control Group:

a) Physically and clinically normal adult above 18 years whose weight is above 36 kg.
b) Their staple dietary source is from local crops grown (at least a part of their diet was from the local crops grown).
c) None of the subjects has a previous history of taking Zn & Mo supplementation in the previous two weeks.

Food grain from residing places of the case and control group from Eastern Cape, South Africa as well as West Bengal, India are taken for evaluation of Zn & Mo content. The staple diet of the people in these areas is maize and rice for RSA and India respectively. Number of samples for food grain analysis is thirty for both RSA and India. Hair samples are collected from all subjects by a doctor and kept in a special container for Zn & Mo analysis.

Zn & Mo are analyzed according to the standard methods of American Public Health Association (American Public Health Association [APHA], 1998). Both food grain and hair sample are repeatedly digested in aqua-regia, then filtered and make up the volume. The filtrate is analyzed by AAS (GBC make 908 Model). A blank is run similarly in each case.

## 3. Results

**Figure 1:**
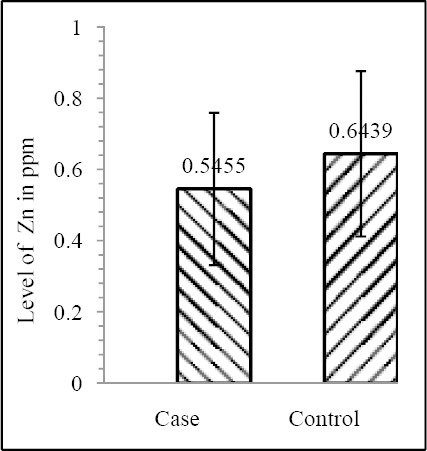
Comparison of level of Zn in hair between the case & control groups in India

**Figure 2:**
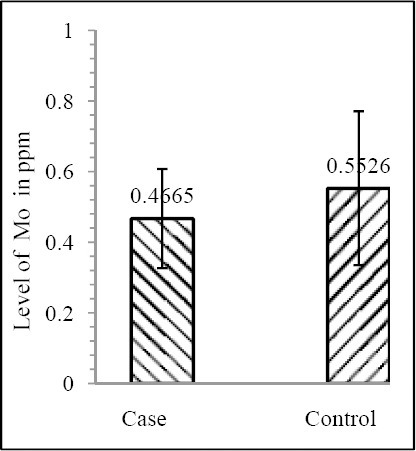
Comparison of level of Mo in hair between the case & control groups in India

**Figure 3:**
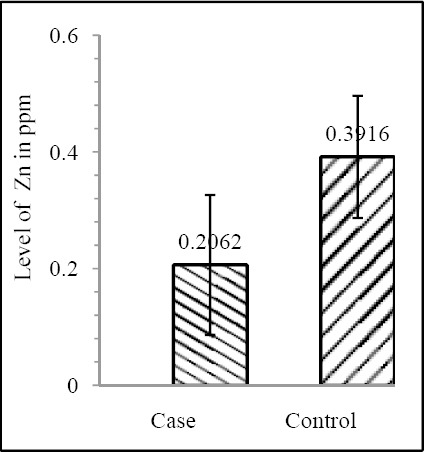
Comparison of level of Zn in hair between the case & control groups in RSA

Our results shows that EC patient of South African case group has significantly reduced level of Zn & Mo in hair with respect to the South African control group (both Zn & Mo: p<0.0001) (Figure 1 & Figure 2; Table 1). In Indian context it is not significant (both Zn & Mo: p≥0.05) (Figure 2 & Figure 3; Table 1). In comparison between the control groups of RSA (hot spot) and India (cold spot) it is found that RSA has significantly reduced level of both Zn & Mo in hair with respect to India (both Zn & Mo: p<0.0001) (Figure 5 & Figure 6; Table 2). Similarly, case group of RSA shows a significantly reduced level of both Zn & Mo in hair in relation to Indian case group (Zn: p<0.0001 & Mo: p<0.005) (Figure 7 & Figure 8; Table 2). Even South African control group has significantly reduced level of both Zn & Mo in hair with respect Indian case group (Zn: p<0.001 & Mo: p<0.00001) (Figure 9 & Figure 10; Table 3).

**Table 1:** Comparison of level (ppm) of Zn & Mo in hair between the case and control groups

Elements	INDIA			RSA		

	Case (Mean ± S.D.)	Control (Mean ± S.D.)	p-value	Case (Mean ± S.D.)	Control (Mean ± S.D.)	p-value
Zn	0.5455±0.2142	0.6439±0.2321	0.082852	0.2062±0.1199	0.3916±0.1047	1.121×10^-8^
Mo	14.929±0.4665	17.682±0.5526	0.065334	0.1975±0.0907	0.3029±0.0791	5.876×10^-6^

S.D.: Standard Deviation

**Figure 4:**
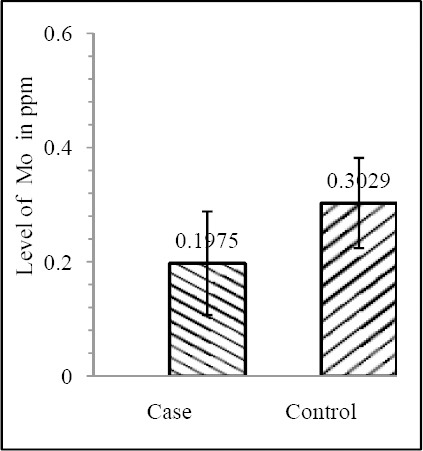
Comparison of level of Mo in hair between the case & control groups in RSA

**Figure 5:**
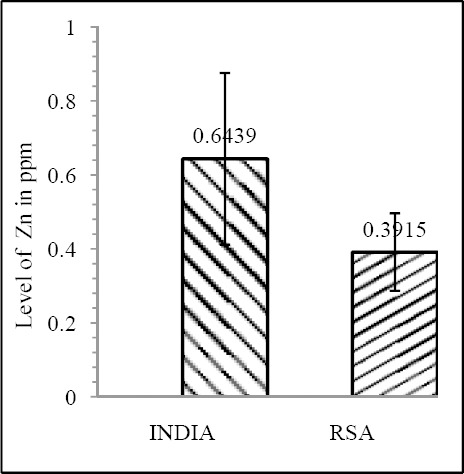
Comparison of level of Zn in hair between the control groups of India & RSA

**Figure 6:**
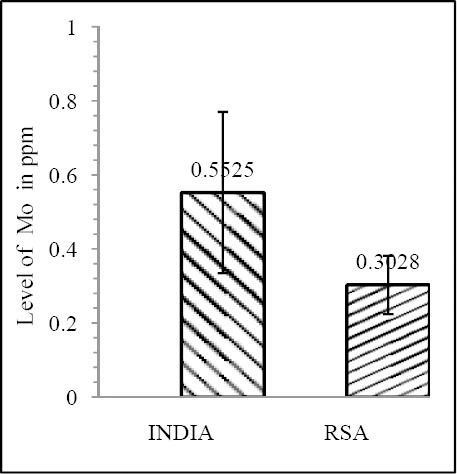
Comparison of level of Mo in hair between the control groups of India & RSA

**Table 2:** Comparison of level (ppm) of Zn & Mo between the control groups (INDIA & RSA) and between the case groups (INDIA & RSA)

Elements	Control			Case		

	India (Mean ± S.D.)	RSA (Mean ± S.D.)	p-value	India (Mean ± S.D.)	RSA (Mean ± S.D.)	p-value
Zn	0.6439±0.2321	0.3916±0.1047	5.079×10^-7^	0.5455±0.2142	0.2062±0.1199	8.175×10^-11^
Mo	0.5526±0.2181	0.3029±0.0791	7.86×10^-8^	0.4665±0.1405	0.1975±0.0907	4.94×10^-3^

S.D.: Standard Deviation

**Figure 7:**
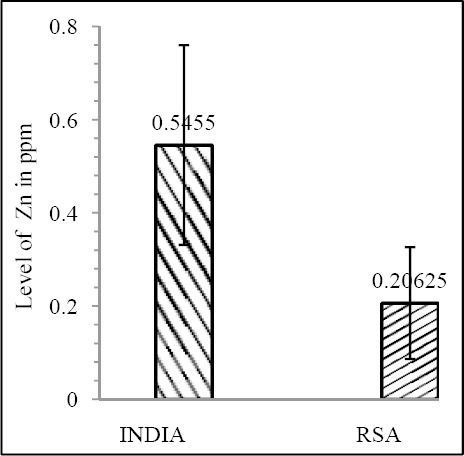
Comparison of level of Zn in hair between the case groups (RSA & India)

**Figure 8:**
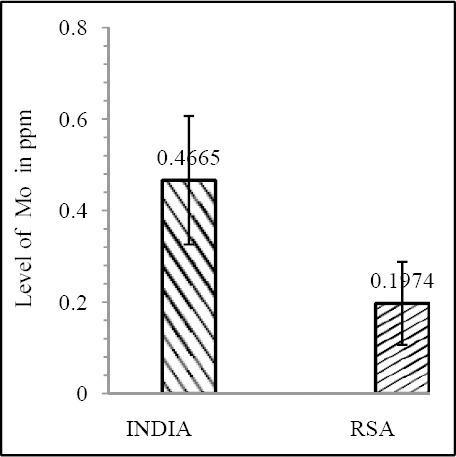
Comparison of level of Mo in hair between the case groups (RSA & India)

**Figure 9:**
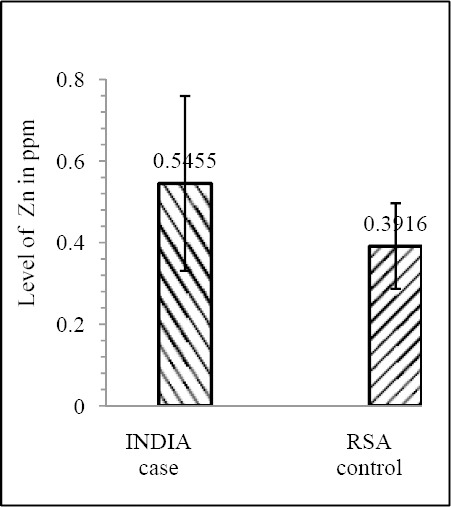
Comparison of level of Zn in hair between the case (India) and control (RSA) groups

**Figure 10:**
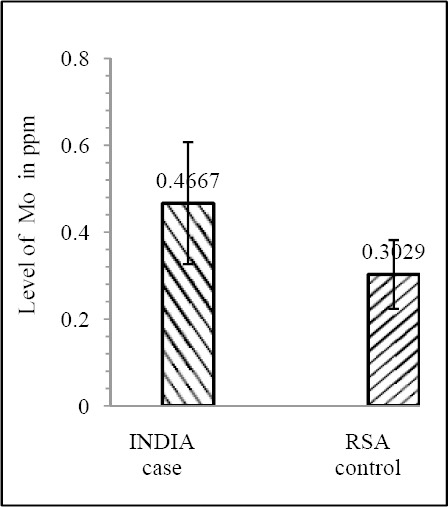
Comparison of level of Mo in hair between the case (India) and control (RSA) groups

**Table 3:** Comparison of level (ppm) of Zn & Mo between the case (India) & the control (RSA) groups

	Level of Zn		Level of Mo	

	Mean ± S.D.	p-value	Mean ± S.D.	p-value
Case (India)	0.5455 ± 0.2142	5.34874×10^-4^	0.4667 ± 0.1405	3.00558×10^-7^
Control (RSA)	0.39156 ± 0.1047		0.3029± 0.07907	

S.D.: Standard Deviation

**Figure 11:**
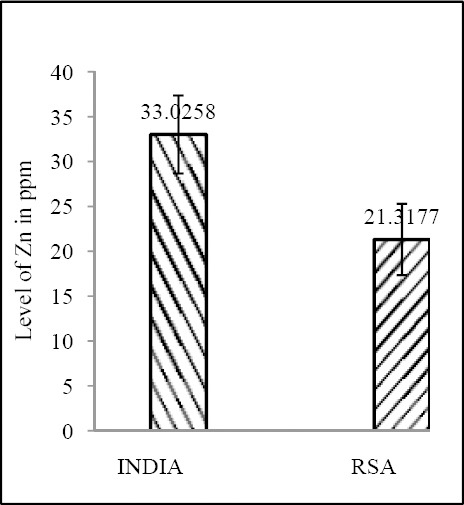
Comparison of level of Zn in food grain between India & RSA

**Figure 12:**
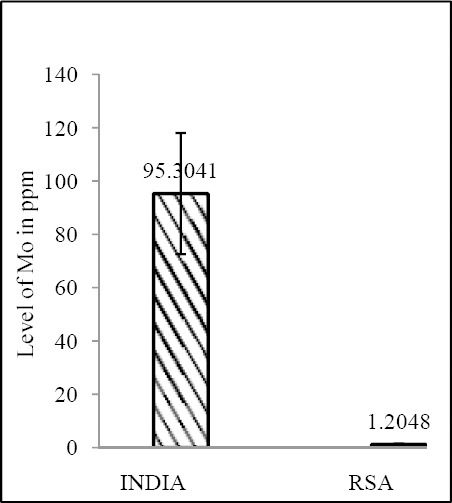
Comparison of level of Mo in food grain between India & RSA

Zn & Mo concentration of food grain in RSA and India shows a correlative pattern (both Zn & Mo: p<0.0001) (Figure 11 & Figure 12; Table 4).

**Table 4:** Comparison of level (ppm) of Zn & Mo in food grain among the India & RSA groups

	Level of Zinc		Level of Mo	

	Mean ± S.D.	p-value	Mean ± S.D.	p-value
India	33.0258 ± 4.3477	4.151 × 10^-16^	95.3042 ± 22.7483	1.76 × 10^-31^
RSA	21.31774 ± 3.9818		1.2048 ± 0.3452	

S.D.: Standard Deviation

## 4. Discussion

All the statistical evaluations are indicating a deficiency of Zn & Mo in hair among RSA population and the deficiencies of Zn & Mo may play a distinct role for the development of EC in Eastern Cape, RSA (hot spot) and this is also supported by other studies (Mellow et al., 1983; Barch et al., 1986; Yang, 1980; Rogers et al., 1993; Luo et al., 1989). The nutritional status of diet plays a significant role in EC. It has been reported that supplement of Zn reduces the incidence of EC of Zn deficient rats (Barch et al., 1992; Fong et al., 1998; Fong & Magee, 1999; Ellwood et al., 1994). Our results indicate that apart from metabolic disorders the deficiencies of Zn & Mo can be correlated to the intake of food grain, deficient in Zn & Mo. However the cause for the development of deficiencies of Zn & Mo in hair of EC patient has been partially answered from our study.
